# A consensus statement for safety monitoring guidelines of treatments for major depressive disorder

**DOI:** 10.3109/00048674.2011.595686

**Published:** 2011-09-03

**Authors:** Seetal Dodd, Gin S Malhi, John Tiller, Isaac Schweitzer, Ian Hickie, Jon Paul Khoo, Darryl L Bassett, Bill Lyndon, Philip B Mitchell, Gordon Parker, Paul B Fitzgerald, Marc Udina, Ajeet Singh, Steven Moylan, Francesco Giorlando, Carolyn Doughty, Christopher G Davey, Michael Theodoras, Michael Berk

**Affiliations:** ^1^School of Medicine, Deakin University, Geelong, Victoria, Australia; Department of Psychiatry, University of Melbourne, Victoria, Australia; ^2^Discipline of Psychiatry, Sydney Medical School, University of Sydney, Sydney, New South Wales, Australia; ^3^Department of Psychiatry, University of Melbourne, Victoria, Australia; ^4^Brain and Mind Research Institute, University of Sydney, Sydney, New South Wales, Australia; ^5^Toowong Specialist Clinic, Level 2/54 Jephson St, Toowong, Brisbane, Queensland, Australia; ^6^School of Psychiatry and Clinical Neurosciences, University of Western Australia, Australia; School of Medicine, University of Notre Dame, Western Australia, Australia; ^7^School of Psychiatry, University of New South Wales, Sydney, Australia; Black Dog Institute, Sydney, Australia; ^8^Monash Alfred Psychiatry Research Centre, Alfred and Monash University School of Psychology and Psychiatry, Melbourne, Victoria, Australia; ^9^Bipolar Disorders Program, Clinical Institute of Neuroscience, Hospital Clinic, University of Barcelona, Institut d'Investigacions Biomèdiques August Pi i Sunyer (IDIBAPS), Centro de Investigación Biomédica en Red de Salud Mental (CIBERSAM), Barcelona, Catalonia, Spain; ^10^School of Medicine, Deakin University, Geelong, Victoria, Australia; ^11^Child and Family Specialty Service, Canterbury District Health Board; Department of Public Health and General Practice, University of Otago, Christchurch, New Zealand; ^12^Orygen Youth Health Research Centre, Parkville, Victoria, Australia; ^13^Eating Disorders Program, New Farm Clinic, Brisbane, Queensland, Australia; ^14^School of Medicine, Deakin University, Geelong, Victoria; Department of Psychiatry, University of Melbourne, Victoria; Mental Health Research Institute, Parkville, Victoria; Orygen Youth Health Research Centre, Parkville, Victoria, Australia

## Abstract

**Objective:**

This paper aims to present an overview of screening and safety considerations for the treatment of clinical depressive disorders and make recommendations for safety monitoring.

**Method:**

Data were sourced by a literature search using MEDLINE and a manual search of scientific journals to identify relevant articles. Draft guidelines were prepared and serially revised in an iterative manner until all co-authors gave final approval of content.

**Results:**

Screening and monitoring can detect medical causes of depression. Specific adverse effects associated with antidepressant treatments may be reduced or identified earlier by baseline screening and agent-specific monitoring after commencing treatment.

**Conclusion:**

The adoption of safety monitoring guidelines when treating clinical depression is likely to improve overall physical health status and treatment outcome. It is important to implement these guidelines in the routine management of clinical depression.

Major depressive disorder (MDD) afflicts an estimated 16% to 20% of the population during their lifetime [1-3]. In Australia 3.1% of men and 5.1% of women are affected in any 12-month period [4]. Current treatments include pharmacological, psychological and physical therapies. Antidepressants are amongst the most commonly prescribed classes of drugs [5] and their use continues to grow [6]. Adverse outcomes are part of the landscape in prescribing medications and therefore management of safety issues need to be an integral part of practice.

The adverse effects of antidepressants vary between individual drugs and drug classes and have been extensively documented [7-9]. In general tricyclic antidepressants (TCAs) and monoamine oxidase inhibitors (MAOIs) are associated with more severe adverse effect profiles than the newer antidepressants. MAOIs interact with dietary tyramine necessitating patient counselling about dietary risks [10]. While there is substantial intra-variation in tolerability, MAOIs and TCAs retain an important place in the management of refractory depression [10] and the more putatively biological depressive disorders such as melancholia [11]. The selective serotonin reuptake inhibitors (SSRIs) and selective serotonin and noradrenaline reuptake inhibitors (SNRIs) broadly have lower toxicity than older agents [12], but have also been associated with significant adverse effects including cardiovascular effects, sexual dysfunction [8], weight gain [13], electrolyte disturbances, haematological effects [8] and hepatic damage [14]. A possible increase in suicidal thoughts and self-destructive behaviour in paediatric patients prescribed antidepressants has been documented [15] and regulatory warnings have been extended for young people up to the age of 25 years [16]. Most treatment guidelines recommend clinician vigilance for adverse symptoms or caution when administering antidepressants to people from high risk populations, including those with a prior history of medication sensitivity, the medically unwell [17], the elderly [18], adolescents [19] and pregnant or breastfeeding women [20]. Specific recommendations are, however, not widely available.

MDD is frequently comorbid with physical problems and illnesses including obesity [21], cardiovascular disease and diabetes mellitus [22], substance misuse and other mental disorders, reflecting both antecedent and consequence pathways [23]. This may affect the efficacy of treatments for MDD as well as increasing the vulnerability of patients to adverse effects and risk of harmful drug interactions.

Non-pharmacological treatments for MDD including psychotherapy [24] have also been associated with adverse events. Such adverse effects are, however, less frequently reported. Complementary and alternative treatments can be associated with adverse events [25] and pharmacological interactions. Even exercise, which has an established evidence base in depression treatment [26], may be associated with adverse effects in individuals with comorbid cardiovascular disease, prolonged QT interval or who attempt strenuous activity without a period of adjustment [27]. However, gradual exercise training for rehabilitation following a major cardiac event in depressed individuals is associated with 73% reduced mortality when compared to depressed individuals who did not participate in exercise training [28].

Guidelines exist for safety monitoring of treatments for schizophrenia [29], bipolar disorder [30] and for specific medications (e.g. clozapine) [31], but there are none for clinical depression. Previous Australian guidelines focus on treatment algorithms [32]. Guidelines for baseline screening and safety monitoring of antidepressant treatments may significantly reduce risks of adverse events.

## Method

Data were sourced by a literature search using MEDLINE and a manual search of scientific journals to identify relevant articles. Searches were conducted on multiple occasions by individual co-authors. Draft guidelines were prepared and serially revised in an iterative manner until all co-authors gave final approval of content. The process was completed over an 18 month period in 2010 and 2011.

### The decision to treat

The management of depression should include an appropriate diagnostic work up, especially to exclude organic or self-limiting determinants. A person's individual risk, prior treatment history and therapeutic preferences should be assessed prior to treatment selection.

Choice of treatment must carefully balance efficacy, potential harms and patient preference, facilitating an informed choice for patients to treatment that best matches their presentation [33]. The following sections will address evidence-based pre-treatment screening and monitoring of treatment of depression and are shown in Table 1. Baseline screening and monitoring recommendations for specific depression augmentation strategies (e.g. lithium [30], the atypical antipsychotics [29]) are reviewed extensively elsewhere and hence are not included below. Similarly, monitoring of psychological therapy is beyond the scope of this paper [24].

**Table 1:** Monitoring recommendations for patients treated for major depressive disorder

Monitoring parameter	Agent	Frequency	Comments
Electrocardiogram for QT prolongation	Tricylic antidepressants	Baseline, after initial dose titration and at dose changes	More caution is indicated in children and the elderly and at higher doses
Liver function test	Agents with hepatic liability	At the start of treatment and at 6, 12 and 24 weeks of treatment and when clinically indicated	Mandated for agomelatine, other agents as indicated
BMI and waist circumference	Agents with known weight gain liability	Baseline At one month and at 6 month intervals	More caution with mirtazapine, mianserin, tricyclics and MAOIs
Vitamin D, B12, folate, zinc, magnesium	All antidepressants	Baseline	If indicated
Electrolytes for hyponatraemia	SSRIs, mirtazapine, SNRIs and TCAs	At baseline and after one month if clinically indicated in high risk groups, especially the elderly (> 65 years)	More frequent monitoring in elderly or those with existing hyponatraemia, follow up testing of urine and serum osmolality
Full Blood Examination to detect neutropaenia and thrombocytopaenia	Mirtazapine and mianserin	If clinically indicated	
Suicidality	All antidepressants, especially SSRIs in adolescents	Weekly in the first few weeks of treatment. More frequent monitoring may be required in more severely depressed or suicidal patients	Particular caution in adolescents Warn next of kin/carer and ask for their collateral monitor as well
Antidepressant side-effect monitoring or Checklist	All antidepressants	At one month and with reviews	
Bone mineral density	SSRIs	As clinically indicated in high risk groups	
Blood pressure monitoring	Venlafaxine, tricyclics and MAOIs	With venlafaxine, tricyclics and MAOIs; at baseline, and with significant dose increase and 3-6 monthly after stabilization	Closer monitoring of MAOI's in first weeks (tolerance occurs)

### Baseline screening prior to commencement of treatment

#### Patient history and physical examination

Obtaining a thorough patient history and examination prior to commencing treatment is important in order to (i) determine whether medical factors may be influencing the current presentation, (ii) determine and document any risk factors which may increase susceptibility to adverse outcomes, (iii) establish baseline measures of physical and mental health in order to monitor treatment efficacy and safety, and (iv) clarify depressive type and assist treatment selection.

Specific medical history information that can impact on monitoring and influence outcome includes substance use (e.g. alcohol [34], tobacco [35,36] and prescribed and illicit drugs), physical activity [37,38], life events, diet [39,40] and chronic and medical illness [41] as well as family history, history of illness and treatment history [42]. Personal or family history of thyroid, cardiac and cerebrovascular disease, hypertension, dyslipidaemia and/or diabetes mellitus should alert clinicians to increased risks associated with antidepressant treatments, and guide targeted baseline assessments.

Although uncommon, organic causes of depressive symptoms should be considered and evaluated before treatment is initiated. Organic causes include metabolic disorders, some vitamin deficiencies (e.g. vitamin D [43, 44], folate, B12, magnesium [45], zinc [46]), chronic pain, sleep apnoea, cancer, cerebrovascular disease, neurological disorders, traumatic brain injury, autoimmune disease (systemic lupus erythematosus), some infections (hepatitis and HIV) and endocrine disorders [47]. Thyroid disease is an occasional medical cause of depressive symptom onset or persistence whilst Cushing's and Addison's disease are rare causes [48].

#### Blood and serological testing

Some organic causes of depressive symptoms can be detected through routine blood screening. The following tests may have a role in baseline screening prior to anti-depressant treatment.

#### Thyroid stimulating hormone (TSH) and thyroid hormones

Hyperthyroidism and hypothyroidism can be associated with mood disorders. Symptoms of hyperthyroidism include dysphoria, anxiety, restlessness, emotional lability and impaired concentration. Symptoms of hypothyroidism include psychomotor retardation, decreased appetite, fatigue and lethargy, which when severe mimics melancholic depression [48]. Subclinical and past thyroid dysfunction is a risk factor for depression and antidepressant non-response [49]. Identification of abnormal thyroid function in depressed patients should prompt correction of thyroid hormone levels (T3 and T4), and where necessary, referral to an endocrinologist for further investigation. Depressive symptoms may resolve with correction of thyroid hormone levels. However, for some patients depressive symptoms may persist and require additional treatment.

#### Full blood examination (FBE)

Full blood examination (FBE) can identify anaemia, potential underlying infections and blood dyscrasias that may contribute to depressive symptoms. Anaemia can induce fatigue and may be misinterpreted as, or contribute to, depressive symptoms. Alterations in leucocytes may alert to the presence of an underlying infection or other pathological process (e.g. cancer).

#### Liver function tests

Diseases of the liver have been associated with depression [50], and some antidepressant treatments are associated with hepatotoxicity [14]. Impaired liver function at baseline should prompt further investigation to determine and treat any hepatic disease. High levels of GGT and AST may suggest comorbid alcohol abuse, especially if concurrent with an increased mean cell volume [51]. Intravenous drug use may result in hepatitis C and cause abnormal liver function tests (LFT).

#### Screening for viral and other infections

Viral infections including human immunodeficiency virus (HIV) [52], hepatitis C [53], West Nile virus [54] and Epstein-Barr virus [55, 56] are associated with depression, albeit uncommonly. This may relate directly to the infection or be consequent to elevated levels of cytokines that may themselves be biomarkers of risk for depression [57]. Fatigue is common following acute infective illness [58] and may be mistakenly attributed as a depressive symptom. In addition, antiviral treatment, most notably PEGylated interferon, can cause episodes of depression [59]. Though uncommon in contemporary practice, non-viral infections such as syphilis [60] can also cause depressive symptoms. Fatigue and depressive symptoms may be a response to activation of the immune system [61], suggesting that broader associations between infections, treatments for infections and the manifestation of depressive symptoms may exist. Prior to commencing antidepressants, a detailed history of past infectious illness and treatments should be acquired, and supplemented with serological testing and testing of inflammatory markers (erythrocyte sedimentation rate (ESR) and C-reactive protein (CRP)) if current infection is suspected.

#### Alcohol and illicit substance screen

Alcohol abuse or dependence are causal factors contributing to depression [62]. Many other substances have also been associated with depression; however, the direction of causality has not been clearly determined. Smoking can increase the risk of depression [35], through direct neurotransmitter mechanisms and via pharmacokinetic drug interactions (e.g. tar components inducing hepatic isoenzymes CYP1A1, CYP1A2 and possibly CYP2E1) and pharmacodynamic drug interactions with nicotine [63]. Depressive symptoms may also emerge with smoking cessation [64]. Cannabis, amphetamine and cocaine use are also associated with depression, and intoxication or withdrawal can be confused with a mood disorder [65]. Furthermore, comorbid alcohol or substance abuse are associated with antidepressant treatment resistance and non-adherence [66]. Obtaining a detailed drug and alcohol history is essential in patients with depression, although clinicians should be cognisant of potential underreporting. Blood or urine screening may be useful in selective cases although such screening only identifies substances present at the time of the test. Collateral information from relatives and friends, as well as other health professionals is invaluable to fully explore substance and alcohol issues. Raised transaminases, elevated carbohydrate deficient transferrin and increased mean cell volume are a clue as to the presence of alcohol abuse.

#### Screen for prescription medications

Several medications are known to induce depressive symptoms. Interferon and other immune active agents (previously mentioned) may be directly causative of depressive episodes [59]. Benzodiazepine use may be associated with depression [67]. Opiates are frequently utilized agents especially in the context of chronic pain. Depression and chronic pain are commonly comorbid and iterative, and it is possible that chronic opiate use has a noxious effect on mood [68,69]. Other potential mood modifying agents include some steroidal and other hormonal treatments [70,71], cardiovascular drugs, cholesterol lowering agents, parkinsonian agents, antibiotic agents [72], antineoplastic agents [73], anticonvulsants [74], typical antipsychotics and analgesics [75-78]. If there is a temporal association of the onset of depression and the commencement of a medication, then an assumption of causality is practicable.

#### Vitamin D

A high prevalence of vitamin D insufficiency is found in most western societies [79] particularly amongst the elderly [80-83]. Psychiatric cohorts appear to have even higher rates of Vitamin D insufficiency [43], with 58% of inpatients in one study having vitamin D insufficiency and 11 % showing deficiency. Low levels of vitamin D, which are common in winter, may contribute to seasonal affective disorder (SAD) [84,85] and a higher likelihood of developing a depressed mood [86,87]. There are some data, albeit inconsistent, that vitamin D supplementation may prevent or improve depressive symptoms [88,89]. While a comprehensive picture of the role of vitamin D in depression is yet to emerge, the high yield from screening and the simplicity of supplementation suggests that screening may be worthwhile. There is some evidence that folate, vitamin B12 magnesium and selenium, amongst other nutrients, are deficient in some individuals with depression, and inconsistent data that supplementation may be of value [45,90-92]. The absence of quality data limits evidence-based recommendations regarding screening, but this should be considered if indicated by history.

#### Cognitive neuropsychological testing

Depression often overlaps with dementia and mild cognitive impairment in elderly patients. Late onset depression may be a prodrome of cognitive decline [93] and depressive symptoms can be confused with dementia [94]. In patients with late-onset depression, diagnostic work up may require formal assessment of cognitive impairment. Late onset vascular depression has been associated with brain small vessel disease demonstrated by hyperintensities on MRI scans [95]. Patients with consistent symptoms should be assessed for vascular risk factors such as smoking, hypertension and diabetes.

#### Metabolic profile, waist circumference and body mass index

Many antidepressants are associated with weight gain, but depression, particularly atypical depression, can cause weight gain independent of medication [21,96]. Metabolic syndrome is a cluster of risk factors which identify individuals at high risk for cardiovascular disease and type II diabetes mellitus [97]. The prevalence of metabolic syndrome in patients with current major depression was found to be 56% in a Finnish study [98] and 25% in a German study [99]. The relationship between depression and metabolic syndrome is bidirectional, with the syndrome also predictive of increased risk for depression [100]. A study of depressed inpatients recommended baseline metabolic screening for all hospitalized depressed patients and re-screening during remission [99].

Baseline measurements of height, weight (to calculate body mass index) and waist circumference should be undertaken. Patients should be questioned about risk factors for metabolic syndrome including smoking, personal or family history of diabetes, while observational analysis will also commonly suggest those at high risk or likelihood of the syndrome or its complications, such as sleep apnoea. Where appropriate, advice regarding smoking cessation should be given. Guidelines for reviewing risk factors for metabolic syndrome during antidepressant treatment are given in the section detailing ongoing monitoring.

#### Cardiovascular disease

People with depression are at an increased risk of developing cardiovascular disease [101]. In addition, some antidepressants (in particular TCAs) have associated adverse cardiovascular effects, although most of the newer antidepressants demonstrate low cardiotoxicity. TCAs may affect cardiac conduction and should be used with caution in patients with pre-existing cardiac disorders (especially in those with recent myocardial infarction and arrhythmias) [102]. Although rare, case reports of adverse cardiac events with newer antidepressants exist, including atrial fibrillation associated with fluoxetine treatment [102] and mild bradycardia with fluoxetine, fiuvoxamine and paroxetine [12]. Adverse cardiac events usually occur in patients with additional risk factors such as smoking [103]. An ECG is probably indicated at baseline and after attainment of therapeutic dosage in individuals with clinical risk factors who have been prescribed agents that can alter the ECG, particularly TCAs. Baseline monitoring of blood pressure and pulse is useful and particularly important if a MAOI or TCA is prescribed [104]. Venlafaxine can cause tachycardia and increased blood pressure, and this merits monitoring [105]. Postural hypotension is particularly common with the MAOIs early in the treatment course, although tolerance typically develops. Antidepressant treatment may cause changes in cardiac function that are not outside the clinically normal range [12], therefore baseline measurements will be required to detect these changes.

#### Twenty-four-hour urinary free cortisol

Hypercortisolaemia and hypocortisolaemia are both associated with depression, although patients typically present with different symptom clusters. Hypocortisolaemia may be more likely to be associated with ‘atypical’ depression, with symptoms including hypoarousal, hypersomnia, hyperphagia, lethargy, pain, fatigue and relative apathy, whereas hypercortisolaemia may be associated with ‘typical’ symptoms such as hyper-arousal, anxiety, insomnia and loss of appetite [106]. It is unclear whether hypothalamic-pituitary-adrenal (HPA) axis activation is causative of depression and consequently information about plasma cortisol is of limited clinical use. The exception is hypercortisolaemia associated with Cushing's syndrome, where depressive symptoms are common and may improve with treatment of the endocrine disorder [106]. Endocrine and cytokine dysfunction often co-occur [107], which may also influence mood disturbances. A cushin-goid appearance, impaired glucose tolerance and electrolyte disturbance may point to this diagnosis, and a 24-h urinary free cortisol measurement prior to commencing antidepressant therapy may be useful in this scenario.

#### Pregnancy testing

Screening for pregnancy, including detailed history and consideration of a pregnancy test where indicated is important for women of childbearing age. Pregnancy can influence mood, especially when associated with psycho-social stressors such as an absent partner or when the pregnancy is unplanned [108]. Pregnancy also raises issues of potential drug-induced teratogenicity; hence knowledge of pregnancy status is important for guiding treatment choices [20,109,110].

#### Genetic testing

Pharmacogenetics (genotype associations to medication response and tolerability) is an emerging area of promise in the safer and more effective prescription of various medications. Some pharmacogenetic tests are commercially available while others are still at an experimental stage. Typically, the tests either probe for polymorphisms of genes involved in neurotransmitter function or probe for polymorphisms of genes involved in drug metabolism [111]. Currently, the role of genotyping to predict antidepressant adverse effects remains unclear. While there is evidence that genotyping cytochrome P450 polymorphisms detects poor metabolisers and ultra-rapid metabolizers with some level of accuracy, their clinical utility in dose finding remains under investigation [112,113]. At present there is no evidence supporting the use of these genetic tests in routine clinical practice, particularly as there is no demonstrated relationship between blood concentrations of the SSRIs or other new antidepressants and clinical efficacy. The role of blood-brain barrier pump polymorphisms is also being explored [114]. There is limited evidence that certain genotypes are associated with higher rates of emergent suicidality while receiving an antidepressant, but results remain mixed [115]. At this stage, routine genetic testing of patients commencing antidepressants does not appear to have sufficient evidence to guide recommendations. In selected patients with a history of medication sensitivity or resistance, there may be a limited place for CYP450 genotyping. Should robust evidence of predicting pharmacogenetic response, tolerability and safety associations emerge there may be a greater place for such genotyping in the future.

#### Neuroimaging

Neuroimaging has advanced substantially in the past three decades, with current technology permitting the assessment of neurochemical concentrations within the brain, more detailed appreciation of its structure and connections and an intriguing insight into its functionality. However, a lack of a consensus as regards diagnostic criteria and clinical phenotype of depression has meant that the emergence of characteristic neuroimaging features that have the necessary sensitivity and specificity at an individual level is yet to occur [116]. The only exception to this may be the use of MRI in cases of late onset depression to confirm the presence of underlying causative small vessel disease. Late onset depression may at times progress to dementia, and MRI will provide a baseline for future assessments.

#### Screening for electroconvulsive therapy

In most jurisdictions, electroconvulsive therapy (ECT) is subject to specific legislative constraints and treatment needs to respect these limitations [117]. Patients should be assessed, including relevant investigations into their general physical health prior to receiving ECT. Acutely ill patients should only receive ECT as an inpatient, at least until their condition stabilizes, as suicide risk may increase in the early phase of treatment [118]. Investigations should be tailored to the individual patient depending on their history, physical examination and diagnosis. Screening for ECT overlaps considerably with pre-anaes-thetic screening and general recommendations in this document.

Uncontrolled hypertension and cerebral tumours are absolute contraindications to ECT. Blood pressure must be assessed. Cochlear implants are a contraindication, unless removed. Cardiac pacemakers are not a contraindication, but it is essential to know the underlying cardiac rhythm and to liaise with the patient's cardiologist on pacemaker management with ECT. Cardiologist opinion should also be sought with patients who have severe aortic stenosis. Anaesthetic monitoring includes ECG, pO_2_ and pCO_2_, and respiratory rate monitoring of pO_2_ and pCO_2_ as well as pulse, blood pressure, respirations, and conscious state are critical in the recovery area.

#### Biomarkers

An emerging research area is that of biomarkers in depression. New data suggest certain biomarkers, including cytokines such as CRP [128] and leptin [129], may be indicative of risk of development of de-novo depression. Other biomarkers have demonstrated a cross-sectional association with depression, such as measures of oxidative stress and neurotrophins [130]. These markers are currently of research interest only, and while provisional evidence suggests potential roles they may shed light on clinical course and outcome [131], data are too preliminary to merit incorporation in routine clinical screening.

### Ongoing monitoring during antidepressant treatment

#### Suicide

After initiating antidepressant treatment, patients should be monitored for suicide risk. A thorough review of suicide monitoring is beyond the scope of this review, however practice guideline are available elsewhere [119, 120].

#### Obesity and metabolic syndrome

Waist circumference and body mass index should be monitored, particularly in patients treated with antidepres-sants associated with weight gain [121]. It is unclear whether being overweight at baseline is associated with further weight increase with antidepressant exposure. Most studies have found no clear association between antidepressant-induced weight gain and baseline measures [122]. In some cases antidepressant treatment may reduce weight and abdominal fat by decreasing anxiety and regulating dietary patterns [123]. A naturalistic study found that weight gain in the first week of antidepressant treatment was a significant predictor of sustained weight gain [13]. There is limited evidence that ethnicity may influence weight increases with antidepressant treatment [124]. Early detection of weight gain and intervention to prevent sustained weight gain may have significant benefits. Monitoring of the metabolic profile may be prudent at 3, 6 and 12 months, then yearly, in all patients, not just in patients who are obese or overweight. While TCAs, MAOIs, mianserin and mirtazapine are most associated with weight gain [121], problems may arise with other newer antidepressants. Paroxetine may be more likely to cause weight gain than other SSRIs, and bupropion may be less likely than the SSRIs [125]. Particular attention should be given to those patients receiving augmentation or combination pharmacotherapy, especially with atypical antipsychotics [126].

#### Cardiovascular status

Cardiovascular status may need monitoring during antidepressant treatment. Electrocardiography (ECG) is recommended for patients being treated with the older antidepressants, especially in those over 45 years of age and those with cardiovascular disorders [12]. ECG should be considered prior to initiation of a TCA, and at steady state, for monitoring the QTc interval and for development of arrhythmias [127]. It may also be considered prior to commencing treatment with an SNRI in high-risk individuals. QT prolongation is a common finding in the elderly. It can lead to Torsades de Pointes (a re-entrant tachycardia), resulting in ventricular fibrillation and sudden cardiac death. It is possible that such outcomes are under-reported. TCAs are known to cause QT prolongation by autonomic effects and effect on rapid outward potassium currents, resulting in delayed repolarization. Patients who have already been treated with a class I antiarrhythmic agent (Na^+^channel blocking) [12] and those who metabolize tricyclics more slowly (cytochrome CYP2D6 poor metabolizer phenotype) may be at increased risk [128]. Electrolyte disturbances, which can be a consequence of some antidepressants, increase the risk of arrhythmias [129].

SSRIs have also been rarely associated with cases of Torsades de Pointes. There were 20 such cases associated with fluoxetine, with one fatality, reported by WHO adverse drug effect monitoring between 1983 and 1999 [130]. Cases associated with SSRIs, however, remain infrequent, sporadic and often involve overdose or drug interactions. Overdose of tricyclics and SSRIs mimic the appearance of Brugada's syndrome with right bundle branch block and ST elevation [131], suggesting some drugs (including fiuoxetine and amitriptyline) may also block cardiac sodium channels. Baseline ECG is important in excluding familial long QT syndrome and to reveal T or U wave abnormalities. Repeat ECG monitoring of QT prolongation may be indicated when significant dose changes are made withTCAs. ECG monitoring with SSRI treatment may be useful in high risk cases, though this is usually unnecessary.

#### Blood dyscrasias

There are rare reports of blood dyscrasias with antide-pressant administration. Case reports have included mian-serin- or mirtazapine-induced neutropenia [132] and citalopram-induced thrombocytopenia [133]. Agranulo-cytosis induced by newer antidepressants is much less common than that which was reported with tricyclic or tetracyclic antidepressants [8]. Monitoring of the full blood examination in patients treated with antidepressants may be prudent when rechallenging patients with a history of a blood dyscrasia. With mirtazapine and mian-serin, one should be alert for emergent symptoms such as fever, sore throat, stomatitis or other signs of infections. If such symptoms occur, haematological investigations should be undertaken and the treatment stopped. Given the very low yield of screening for this very rare adverse event, routine haematological monitoring is not justified.

#### Hepatotoxicity

Numerous antidepressants have been found to affect the liver. The MAOIs and the TCAs are associated with an appreciable though infrequent risk of hepatotoxicity [134]. However, changes in hepatic enzyme levels have rarely been reported for SSRIs and other newer antidepressants including paroxetine [135-137], sertraline [138,139], fiuoxetine [140,141], mianserin [142], mirtazapine [143,144], bupropi on [145],trazodone [146,147], nefazodone [14], venlafaxine [146], duloxetine [148] and agomelatine [149]. Disentangling these rare reports from baseline-associated factors is difficult. Antidepressant-induced hepatotoxicity is usually an idiosyncratic adverse drug reaction of low prevalence characterized by elevated ALT and sometimes by jaundice. The consequences of antidepressant-induced changes in hepatic function can range from asymptomatic changes [144] to fatal outcomes [145]. It is unclear whether the use of antidepressants may aggravate existing hepatic disease. Pre-existing hepatic disease may alter the metabolic clearance of some antidepressants and may necessitate dose adjustment [134].

It does not appear that susceptibility to antidepressant-induced hepatotoxicity can be predicted by LFT or ALT before commencing antidepressant treatment. Therefore these tests are not used as a baseline screen to monitor risk for developing *de-novo* hepatic injury in response to antidepressant exposure [134]. However, they may be useful for detecting pre-existing hepatic injury and for establishing a baseline for patients before commencing antidepressant treatment and are required before commencing treatment with agomelatine. Antidepressant-induced hepatotoxicity typically occurs within the first few months of treatment.

Nefazodone has been withdrawn from the Australian market as a result of cases of fatal hepatotoxicity [150,151]. Elevations of serum transaminases are documented in clinical trials of agomelatine but these changes usually return to normal levels after discontinuation. The European Medicines Agency has recommended a liver function test for patients treated with agomelatine at the start of treatment and at 6, 12 and 24 weeks of treatment and when clinically indicated [152].

Lucena *et al*. [134] state that hepatotoxicity is an uncommon and unpredictable idiosyncratic reaction, and that there is no evidence that routine monitoring reduces the rate of hepatotoxicity with antidepressants in general.

After a hepatotoxic reaction, if an antidepressant rechallenge is indicated, monthly liver functions test for the first 6 months of treatment, with discontinuation of treatment if ALT values exceed (by three times) the upper limit of the normal range, if bilirubin levels rise, or if signs or symptoms of liver dysfunction are present, are recommended by the authors. If an individual develops increased serum transaminases, tests should be repeated within 48 h. After discontinuation, monitoring can be continued to ensure that liver function returns to the normal range. Rechallenge should be considered with caution, and only after careful consideration of risks and benefits. Adverse reactions to an antidepressant rechallenge are not uncommon [150] and sometimes a more severe antidepressant-induced hepatotoxic reaction may occur following antidepressant rechallenge [139,153].

#### Hyponatraemia

SSRIs and some other antidepressants have been reported to cause hyponatraemia. The incidence rate in the elderly is 4.7 cases/1000 people treated/year [154,155], but is much lower in younger age groups. It should be suspected if an elderly patient becomes confused or develops a frank delirium after commencement of an antidepressant. It may be why a person's mood may inexplicably worsen. Deaths associated with hyponatraemia have been reported [156]. Liu *et al*. [157] reported on 736 cases between 1980 and 1995 associated with fiuoxetine, fluvoxamine, paroxetine and sertraline use. Hyponatraemia has also been reported for citalo-pram [158], escitalopram [159], duloxetine [160], mir-tazapine [161] and reboxetine [162]. It has been suggested that the risk may be greatest for patients who take fiuoxetine compared to other antidepressants [154]. SSRIs can induce isovolemic hyponatraemia through a syndrome of inappropriate anti-diuretic hormone (SIADH) secretion [159]. Hyponatraemia can resolve with antidepressant discontinuation and reoccur with rechallenge [160]. Baseline screening of patients should include an assessment of risk factors for antidepressant-induced hyponatraemia including advanced age (≥65 years) [157], previous history of antidepressant-induced hyponatraemia [160], low body weight [155], thiazide use [163], and the use of other agents that may cause hyponatraemia such as carbamazepine [164]. Anecdotally, female gender [163] may also be a risk factor. Most cases of hyponatraemia occur within the first 10 weeks of initiating antidepressant treatment, with 79% occurring in the first 3 weeks [154]. Regular monitoring of serum sodium in elderly patients has been recommended by some clinicians [154]. SIADH may also cause a worsening of depressive symptoms [154,159] and therefore should be sought and excluded. SIADH should be investigated if a person reports clinical deterioration, cognitive change or dizziness and fatigue after initiation of antidepressant treatment [159]. There is no current protocol for monitoring serum sodium in patients being treated with antidepressants; however, monitoring after 3-4 weeks of treatment should occur in individuals who match the high risk profile [155].

#### Bone mineral density

There are recent data indicating that SSRIs may reduce bone mineral density [165]. Serotonin has a key signalling role in bone cells and influences bone metabolism. Williams *et al*. showed a loss of 6.2% of bone mineral density (BMD) with SSRI treatment [166]. This magnitude of bone loss has the potential to lead to a 20% increased fracture risk. Bolton *et al*. described SSRI use in older adults being associated with an increased odds of osteoporotic fractures (OR = 1.45), as for other mono-amine antidepressants (OR =1.15), although the relationship was weaker [167]. These data are supported by a study showing that the risk of non-vertebral fracture for those over 55 years was two-fold for current users of SSRIs compared with non-users of antidepressants [168]. Preliminary data suggest intra-class differences [169], with citalopram seeming to have the lowest impact on osteoblast and osteoclast function. There are data indicating that, independent of treatment, people with depression have lower bone mineral density and a higher fracture risk, which is likely to be a compound result of lifestyle factors, disease state and iatrogenic treatment effects. These disease-related effects, combined with the effects on treatment, suggest that screening for bone mineral density should be considered in people on long-term SSRIs, and particularly in high risk groups such as the elderly, women and those with other risk factors such as fracture history, history of falls, low vitamin D, hypogonadism, hyperparathyroidism, thyroid dysfunction, systemic inflammatory disorders, and corticosteroid use. Physical inactivity, alcohol use and smoking are shared lifestyle risk factors for both depression and low BMD.

#### Other adverse effects

Antidepressant treatment is commonly associated with milder adverse effects. The Antidepressant Side-Effect Checklist itemizes 21 adverse events; nausea or vomiting, sexual dysfunction, increased appetite, decreased appetite, sweating, increased body temperature, weight gain, dry mouth, drowsiness, insomnia, blurred vision, headache, constipation, diarrhoea, problems with urination, palpitations, orthostatic dizziness, vertigo, tremor, disorientation, and yawning [9]. Such adverse effects are important causes of non-adherence to antidepressant treatment [9] and should therefore be routinely monitored.

#### Drug-drug interactions

Monitoring should take into account that antidepressants may interact with other medications and reduce the therapeutic effect or increase side-effects. Perhaps one of the most important differences between the newer antidepressants is their varying potential to cause drug-drug interactions through inhibition of cytochrome-P450 (CYP) enzymes [170]. Fluvoxamine, a potent inhibitor of CYP1A2 and CYP2C19, is a particular cause for concern with clinically relevant interactions reported with caffeine, clozapine, benzodiazepines, warfarin and other medications [170,171]. SSRIs such fluoxetine and paroxetine, which are potent inhibitors of CYP2D6, can also produce relevant interactions with other medications including beta-blockers, tricyclic antidepressants and antipsychotics such as perphenazine [172]. Special attention should be given to fluoxetine because inhibitory effects can persist for up to five weeks after discontinuation. Sertraline, citalopram and escitalopram seem to have less interaction potential than the other SSRIs [170,173]. Duloxetine andbupropion are moderate inhibitors of CYP2D6, while venlafaxine, mirtazapine and reboxetine are weak inhibitors and are less likely to interact with co-administered medications [173]. These factors should be taken into account when starting an antidepressant and in some cases may require dose adjustment.

## Discussion

Choosing a treatment that offers the optimal risk-benefit ratio for a given individual is typically complex. The balance between the patient's prior history of treatment response and tolerability, their current clinical profile and needs, their medical status and personal risks, and the differential tolerability profiles of the drug choices and their efficacy data need to be struck. Optimal safety monitoring is part of a system of care committed to a culture of patient safety. The treating clinician is primarily responsible for decisions regarding treatment choice, making these decisions on the basis of shared decision making, in the context of a collaborative alliance, and communicating the risks and benefits to the patient. In divergent settings, the monitoring arrangements need to vary. Computerized electronic medical records or checklists with reminder prompts may help with safety monitoring; however, systems for lines of communication, co-ordination of monitoring practices and for the interpretation of results need to be developed. If adverse events emerge, the re-balanced risk-benefit ratio needs to be evaluated on an individual basis. Decisions regarding further investigations, referrals to specialist care or the need for treatment changes will need to be made.

Safety monitoring guidelines are developed from indirect data, which are frequently limited and drawn from clinical trial data and surveillance monitoring. Almost no direct studies are available on the utility, cost-benefit ratios, yield or practical implementation of safety monitoring guidelines. The guidelines themselves are the product of an imperfect process of literature review moulded by consensus opinion. They therefore need to be used flexibly and should be moulded by clinical acumen. The recommendations should be adapted to the needs of individual patients, taking into account broad risk factors such as age, general health, comorbidity, polypharmacy, capacity for adherence to monitoring procedures, and clinical diagnosis. It needs to be stressed that these guidelines do not represent a fixed standard of care in a medico-legal context. The principal goal of these guidelines is to increase the probability of prompt detection and intervention for relatively insidious but clinically significant adverse events. They are likely to evolve with further research on risk, tolerability and safety of treatments.

## References

[1] Heo M, Murphy CF, Fontaine KR, Bruce ML, Alexopoulos GS (2008). Population projection of US adults with lifetime experience of depressive disorder by age and sex from year 2005 to 2050. Int J Geriatr Psychiatry.

[2] Kessler RC, Berglund P, Demler O, Jin R, Koretz D, Merikangas KR, Rush AJ, Walters EE, Wang PS (2003). The epidemiology of major depressive disorder: results from the National Comorbidity Survey Replication (NCS-R). JAMA.

[3] Wittchen HU, Jacobi F (2005). Size and burden of mental disorders in Europe: a critical review and appraisal of 27 studies. Eur Neuropsychopharmacol.

[4] Slade T, Johnston A, Oakley Browne MA, Andrews G, Whiteford H (2009). 2007 National Survey of Mental Health and Wellbeing: methods and key findings. Aust N Z J Psychiatry.

[5] Worthy KC, Governale LA Trends in outpatient prescription usage in the U.S., 2002 - 2005.

[6] Hollingworth SA, Burgess PM, Whiteford HA (2010). Affective and anxiety disorders: prevalence, treatment and antidepressant medication use. Aust N Z J Psychiatry.

[7] Wille SM, Cooreman SG, Neels HM, Lambert WE (2008). Relevant issues in the monitoring and the toxicology of antidepressants. Crit Rev Clin Lab Sci.

[8] Mago R, Mahajan R, Thase ME (2008). Medically serious adverse effects of newer antidepressants. Curr Psychiatry Rep.

[9] Uher R, Farmer A (2009). Adverse reactions to anti depressants. Br J Psychiatry.

[10] Stahl SM, Felker A (2008). Monoamine oxidase inhibitors: a modern guide to an unrequited class of antidepressants. CNS Spectr.

[11] Parker G, Fink M (2010). Issues for DSM-5: whither melancholia? The case for its classification as a distinct mood disorder. Am J Psychiatry.

[12] Pacher P, Kecskemeti V (2004). Cardiovascular side effects of new anti depressants and antipsychotics: new drugs, old concerns?. Curr Pharm Des.

[13] Himmerich H, Schuld A, Haack M, Kaufmann C, Pollmacher T (2004). Early prediction of changes in weight during six weeks of treatment with antidepressants. J Psychiatr Res.

[14] Carvajal Garcia-Pando A, Garcia del Pozo J, Sanchez AS, Velasco MA, Rueda de Castro AM, Lucena MI (2002). Hepatotoxicity associated with the new antidepressants. J Clin Psychiatry.

[15] Culpepper L, Davidson JR, Dietrich AJ, Goodman WK, Kroenke K, Schwenk TL (2004). Suicidality as a possible side effect of antidepressant treatment. J Clin Psychiatry.

[16] Leon AC (2007). The revised warning for antidepressants and suicidality: unveiling the black box of statistical analyses. Am J Psychiatry.

[17] Franco-Bronson K (1996). The management of treatment-resistant depression in the medically ill. Psychiatr Clin North Am.

[18] Sommer BR, Fenn H, Pompei P, DeBattista C, Lembke A, Wang P, Flores B (2003). Safety of antidepressants in the elderly. Expert Opin Drug Saf.

[19] Berk M, Dodd S (2005). Antidepressants and adolescents: the bipolar confound?. Hum Psychopharmacol.

[20] Wisner KL, Sit DK (2009). Major depression and anti-depressant treatment: impact on pregnancy and neonatal outcomes. Am J Psychiatry.

[21] Williams LJ, Pasco JA (2009). Lifetime psychiatric disorders and body composition: a population-based study. J Affect Disord.

[22] Turvey CL, Schultz SK, Beglinger L, Klein DM (2009). A longitudinal community-based study of chronic illness, cognitive and physical function, and depression. Am J Geriatr Psychiatry.

[23] Maes M, Leonard B (2011). (Neuro)inflammation and neuroprogression as new pathways and drug targets in depression: From antioxidants to kinase inhibitors. Prog Neuropsychopharmacol Biol Psychiatry.

[24] Berk M, Parker G (2009). The elephant on the couch: side-effects of psychotherapy. Aust N Z J Psychiatry.

[25] Breidenbach T, Hoffmann MW, Becker T, Schlitt H, Klempnauer J (2000). Drug interaction of St John's wort with cyclosporin. Lancet.

[26] Mead GE, Morley W, Campbell P, Greig CA, McMurdo M, Lawlor DA (2009). Exercise for depression. Cochrane Database Syst Rev.

[27] Albert CM, Mittleman MA, Chae CU, Lee IM, Hennekens CH, Manson JE (2000). Triggering of sudden death from cardiac causes by vigorous exertion. N Engl J Med.

[28] Milani RY, Lavie CJ (2007). Impact of cardiac rehabilitation on depression and its associated mortality. Am J Med.

[29] Marder SR, Essock SM (2004). Physical health monitoring of patients with schizophrenia. Am J Psychiatry.

[30] Ng F, Mammen OK (2009). The International Society for Bipolar Disorders (ISBD) consensus guidelines for the safety monitoring of bipolar disorder treatments. Bipolar Disord.

[31] Berk M, Fitzsimons J (2007). Monitoring the safe use of clozapine: a consensus view from Victoria, Australia. CNS Drugs.

[32] Ellis P (2004). Australian and New Zealand clinical practice guidelines for the treatment of depression. Aust N Z J Psychiatry.

[33] Malhi GS, Adams D (2009). Clinical practice recommendations for depression. Ada Psychiatr Scand Suppl.

[34] Coulson CE, Williams LJ (2010). Patterns of alcohol use and associated physical and lifestyle characteristics according to new Australian guidelines. Aust N Z J Psychiatry.

[35] Pasco JA, Williams LJ (2008). Tobacco smoking as a risk factor for major depressive disorder: population-based study. Br J Psychiatry.

[36] Berk M, Ng F (2008). Going up in smoke: tobacco smoking is associated with worse treatment outcomes in mania. J Affect Disord.

[37] Jacka FN, Pasco JA (2011). Lower levels of physical activity in childhood associated with adult depression. J Sci Med Sport.

[38] Pasco JA, Williams LJ (2010). Habitual physical activity and the risk for depressive and anxiety disorders among older men and women. Int Psychogeriatr.

[39] Jacka FN, Kremer PJ (2010). Associations between diet quality and depressed mood in adolescents: results from the Australian Healthy Neighbourhoods Study. Aust N Z J Psychiatry.

[40] Jacka FN, Pasco JA (2010). Association of western and traditional diets with depression and anxiety in women. Am J Psychiatry.

[41] Batterham PJ, Christensen H, Mackinnon AJ (2009). Modifiable risk factors predicting major depressive disorder at four year follow-up: a decision tree approach. BMC Psychiatry.

[42] Zuithoff NP, Vergouwe Y (2009). A clinical prediction rule for detecting major depressive disorder in primary care: the PRE-DICT-NL study. Fam Pract.

[43] Berk M, Jacka FN, Williams LJ, Ng F, Dodd S, Pasco JA (2008). Is this D vitamin to worry about? Vitamin D insufficiency in an inpatient sample. Aust N Z J Psychiatry.

[44] Berk M, Sanders KM (2007). Vitamin D deficiency may play a role in depression. Med Hypotheses.

[45] Jacka FN, Overland S, Stewart R, Tell GS, Bjelland I, Mykletun A (2009). Association between magnesium intake and depression and anxiety in community-dwelling adults: the Hordaland Health Study. Aust N Z J Psychiatry.

[46] Szewczyk B, Poleszak E (2008). Antidepressant activity of zinc and magnesium in view of the current hypotheses of antidepressant action. Pharmacol Rep.

[47] Robertson MM, Katona CLE (1997). Depression and physical illness.

[48] Bauer M, Goetz T, Glenn T, Whybrow PC (2008). The thyroid-brain interaction in thyroid disorders and mood disorders. J Neuroendocrinol.

[49] Fountoulakis KN, Kantartzis S (2006). Peripheral thyroid dysfunction in depression. World J Biol Psychiatry.

[50] Carta MG, Hardoy MC (2007). Association of chronic hepatitis C with major depressive disorders: irrespective of interferon-alpha therapy. Clin Pract Epidemol Ment Health.

[51] Liangpunsakul S, Qi R, Crabb DW, Witzmann F (2010). Relationship between alcohol drinking and aspartate aminotransferase:alanine aminotransferase (AST:ALT) ratio, mean corpuscular volume (MCV), gamma-glutamyl transpeptidase (GGT), and apolipoprotein A1 and B in the U.S. population. J Stud Alcohol Drugs.

[52] Shacham E, Nurutdinova D, Satyanarayana V, Stamm K, Overton ET (2009). Routine screening for depression: identifying a challenge for successful HIV care. AIDS Patient Care STDS.

[53] Bailey DE, Landerman L (2009). Uncertainty, symptoms, and quality of life in persons with chronic hepatitis C. Psychosomatics.

[54] Murray KO, Resnick M, Miller V (2007). Depression after infection with West Nile virus. Emerg Infect Dis.

[55] Miller AH, Silberstein C (1986). Epstein-Barr virus infection and depression. J Clin Psychiatry.

[56] Miller GE, Freedland KE, Duntley S, Carney RM (2005). Relation of depressive symptoms to C-reactive protein and pathogen burden (cytomegalovirus, herpes simplex virus, Epstein-Barr virus) in patients with earlier acute coronary syndromes. Am J Cardiol.

[57] Berk M, Wadee AA, Kuschke RH, O'Neill-Kerr A (1997). Acute phase proteins in major depression. J Psychosom Res.

[58] Bennett BK, Hickie IB (1998). The relationship between fatigue, psychological and immunological variables in acute infectious illness. Aust N Z J Psychiatry.

[59] Martin-Santos R, Diez-Quevedo C (2008). De novo depression and anxiety disorders and influence on adherence during PEGinterferon-alpha-2a and ribavirin treatment in patients with hepatitis C. Aliment Pharmacol Ther.

[60] Dewhurst K (1969). The neurosyphilitic psychoses today. A survey of 91 cases. Br J Psychiatry.

[61] Ortega-Hernandez OD, Shoenfeld Y (2009). Infection, vaccination, and autoantibodies in chronic fatigue syndrome, cause or coincidence?. Ann NYAcad Sd.

[62] Fergusson DM, Boden JM, Horwood LJ (2009). Tests of causal links between alcohol abuse or dependence and major depression. Arch Gen Psychiatry.

[63] Kroon LA (2007). Drug interactions with smoking. Am J Health Syst Pharm.

[64] Aubin HJ (2009). Management of emergent psychiatric symptoms during smoking cessation. Curr Med Res Opin.

[65] Kaminer Y, Connor DF, Curry JF (2008). Treatment of comorbid adolescent cannabis use and major depressive disorder. *Psychiatry* (Edgmont).

[66] Kornstein SG, Schneider RK (2001). Clinical features of treatment-resistant depression. J Clin Psychiatry.

[67] Manthey L, van Veen T (2011). Correlates of (inappropriate) benzodiazepine use: the Netherlands Study of Depression and Anxiety (NESDA). Br J Clin Pharmacol.

[68] Peles E, Schreiber S, Naumovsky Y, Adelson M (2001). Depression in methadone maintenance treatment patients: rate and risk factors. J Affect Disord.

[69] Aharonovich E, Nguyen HT, Nunes EV (2001). Anger and depressive states among treatment-seeking drug abusers: testing the psychopharmacological specificity hypothesis. Am J Addict.

[70] Bahrke MS, Yesalis CE, Wright JE (1996). Psychological and behavioural effects of endogenous testosterone and anabolic-androgenic steroids. An update. Sports Med.

[71] Frye CA (2009). Steroids, reproductive endocrine function, and affect. A review. Minerva Ginecol.

[72] Sternbach H, State R (1997). Antibiotics: neuropsychiatric effects and psychotropic interactions. Harv Rev Psychiatry.

[73] Schaefer M, Engelbrecht MA (2002). Interferon alpha (IFNalpha) and psychiatric syndromes: a review. Prog Neuropsychopharmacol Biol Psychiatry.

[74] Kanner AM, Wuu J, Faught E, Tatum WOt, Fix A, French JA (2003). A past psychiatric history may be a risk factor for topiramate-related psychiatric and cognitive adverse events. Epilepsy Behav.

[75] Hollister LE (1986). Drug-induced psychiatric disorders and their management. Med Toxicol.

[76] Johnson DA (1981). Drug-induced psychiatric disorders. Drugs.

[77] Flaherty JA (1979). Psychiatric complications of medical drugs. J Fam Pract.

[78] Whitlock FA (1981). Adverse psychiatric reactions to modern medication. Aust N Z J Psychiatry.

[79] Mithal A, Wahl D (2009). Global vitamin D status and determinants of hypovitaminosis D. Osteoporos Int.

[80] Pasco J, Henry M, Nicholson G, Sanders K, Kotowicz M (2001). Vitamin D status of women in the Geelong Osteoporosis Study: association with diet and casual exposure to sunlight. Med J Aust.

[81] van der Mei I, Ponsonby A-L (2007). The high prevalence of vitamin D insufficiency across Australian populations is only partly explained by season and latitude. Environ Health Perspect.

[82] McGrath J, Kimlin M, Saha S, Eyles D, Parisi A (2001). Vitamin D insufficiency in south-east Queensland. Med J Aust.

[83] Park S, Johnson M (2005). Living in low-latitude regions in the United States does not prevent poor vitamin D status. Nutr Rev.

[84] Alexopoulos GS, Meyers BS, Young RC, Campbell S, Silbersweig D, Charlson M (1997). ‘Vascular depression’ hypothesis. Arch Gen Psychiatry.

[85] McGrath J (1999). Hypothesis: is low prenatal vitamin D a risk-modifying factor for schizophrenia?. Schrizophr Res.

[86] Milaneschi Y, Shardell M (2010). Serum 25-hydroxyvitamin D and depressive symptoms in older women and men. J Clin Endocrinol Metab.

[87] Ganji V, Milone C, Cody MM, McCarty F, Wang YT (2010). Serum vitamin D concentrations are related to depression in young adult US population: the Third National Health and Nutrition Examination Survey. Int Arch Med.

[88] Khajehei M, Abdali K, Parsanezhad ME, Tabatabaee HR (2009). Effect of treatment with dydrogesterone or calcium plus vitamin D on the severity of premenstrual syndrome. Int J Gynaecol Obstet.

[89] Sanders KM, Stuart AL (2011). Annual high-dose vitamin D3 and mental well-being: randomised controlled trial. Br J Psychiatry.

[90] Gariballa S, Forster S (2007). Effects of dietary supplements on depressive symptoms in older patients: a randomised double-blind placebo-controlled trial. Clin Nutr.

[91] Walker JG, Mackinnon AJ (2010). Mental health literacy, folic acid and vitamin B12, and physical activity for the prevention of depression in older adults: randomised controlled trial. Br J Psychiatry.

[92] Roberts SH, Bedson E (2007). Folate augmentation of treatment - evaluation for depression (FolATED): protocol of a randomised controlled trial. BMC Psychiatry.

[93] Schweitzer I, Tuckwell V, O'Brien J, Ames D (2002). Is late onset depression a prodrome to dementia?. Int J Geriatr Psychiatry.

[94] Wright SL, Persad C (2007). Distinguishing between depression and dementia in older persons: neuropsychological and neuropathological correlates. J Geriatr Psychiatry Neurol.

[95] O'Brien J, Ames D, Chiu E, Schweitzer I, Desmond P, Tress B (1998). Severe deep white matter lesions and outcome in elderly patients with major depressive disorder: follow up study. BMJ.

[96] Cascade E, Kalali AH, Kennedy SH (2009). Real-world data on SSRI antidepressant side effects. *Psychiatry* (Edgmont).

[97] Grundy SM, Cleeman JI (2005). Diagnosis and management of the metabolic syndrome: an American Heart Association/National Heart, Lung, and Blood Institute Scientific Statement. Circulation.

[98] Heiskanen TH, Niskanen LK (2006). Metabolic syndrome and depression: a cross-sectional analysis. J Clin Psychiatry.

[99] Richter N, Juckel G, Assion HJ Metabolic syndrome: a follow-up study of acute depressive inpatients. Eur Arch Psychiatry Clin Neurosci.

[100] Takeuchi T, Nakao M (2009). Association of the metabolic syndrome with depression and anxiety in Japanese men: a 1-year cohort study. Diabetes Metab Res Rev.

[101] Glassman AH, Shapiro PA (1998). Depression and the course of coronary artery disease. Am J Psychiatry.

[102] Murray JB (2000). Cardiac disorders and antidepressant medications. J Psychol.

[103] Khawaja IS, Feinstein RE (2003). Cardiovascular effects of selective serotonin reuptake inhibitors and other novel antidepressants. Heart Dis.

[104] O'Brien S, McKeon P, O'Regan M, O'Flaherty A, Patel R (1992). Blood pressure effects of tranylcypromine when prescribed singly and in combination with amitriptyline. J Clin Psychopharmacol.

[105] Howell C, Wilson AD, Waring WS (2007). Cardiovascular toxicity due to venlafaxine poisoning in adults: a review of 235 consecutive cases. Br J Clin Pharmacol.

[106] Wolkowitz OM, Burke H, Epel ES, Reus VI (2009). Glucocorticoids. Mood, memory, and mechanisms. Ann N Y Acad Sci.

[107] Anisman H, Ravindran AV, Griffiths J, Merali Z (1999). Endocrine and cytokine correlates of major depression and dysthymia with typical or atypical features. Mol Psychiatry.

[108] Marchesi C, Bertoni S, Maggini C (2009). Major and minor depression in pregnancy. Obstet Gynecol.

[109] Dodd S, Berk M (2006). The safety of medications for the treatment of bipolar disorder during pregnancy and the puerperium. Curr Drug Saf.

[110] Dodd S, Berk M (2004). The pharmacology of bipolar disorder during pregnancy and breastfeeding. Expert Opin Drug Saf.

[111] Thomas KL, Ellingrod VL (2009). Pharmacogenetics of selective serotonin reuptake inhibitors and associated adverse drug reactions. Pharmacotherapy.

[112] de Leon J, Armstrong SC, Cozza KL (2006). Clinical guidelines for psychiatrists for the use of pharmacogenetic testing for CYP450 2D6 and CYP450 2C19. Psychosomatics.

[113] Ingelman-Sundberg M (2004). Pharmacogenetics of cytochrome P450 and its applications in drug therapy: the past, present and future. Trends Pharmacol Sci.

[114] Singh A (2007). Pharmacogenomics: the potential of genetically guided prescribing. Aust Fam Physician.

[115] Laje G, Paddock S (2007). Genetic markers of suicidal ideation emerging during citalopram treatment of major depression. Am J Psychiatry.

[116] Malhi GS, Lagopoulos J (2008). Making sense of neuroimaging in psychiatry. Ada Psychiatr Scand.

[117] Loo C, Trollor J, Alonzo A, Rendina N, Kavess R (2010). Mental health legislation and psychiatric treatments in NSW: electroconvulsive therapy and deep brain stimulation. Australas Psychiatry.

[118] Tiller JWG, Lyndon RW (2003). Electroconvulsive therapy: an Australasian guide.

[119] McDermott B, Baigent M (2010). beyondblue Expert Working Committee (2010) Clinical practice guidelines: Depression in adolescents and young adults. http://www.beyondblue.org.au/index.aspx?link_id=6.1247&tmp=FileDownload&fid=1625.

[120] The Australian Government Department of Health and Ageing (2007). The Living Is For Everyone (LIFE) framework. http://www.livingisforeveryone.com.au/IgnitionSuite/uploads/docs/LIFE_framework-web.pdf.

[121] Risselada AJ, Mulder H, Heerdink ER, Grube AM, Wilmink FW, Egberts TC The association between serotonin 2C receptor polymorphisms and weight gain and eating behavior in patients using mirtazapine: a prospective follow-up study. J Clin Psychopharmacol.

[122] Fernstrom MH, Kupfer DJ (1988). Antidepressant-induced weight gain: a comparison study of four medications. Psychiatry Res.

[123] Hainer V, Kabrnova K, Aldhoon B, Kunesova M, Wagenknecht M (2006). Serotonin and norepinephrine reuptake inhibition and eating behavior. Ann N Y Acad Sci.

[124] Ormerod S, McDowell SE, Coleman JJ, Ferner RE (2008). Ethnic differences in the risks of adverse reactions to drugs used in the treatment of psychoses and depression: a systematic review and meta-analysis. Drug Saf.

[125] Fava M (2000). Weight gain and antidepressants. J Clin Psychiatry.

[126] Seo HJ, Jung YE, Woo YS, Jun TY, Chae JH, Bahk WM (2009). Effect of augmented atypical antipsychotics on weight change in patients with major depressive disorder in a naturalistic setting. Hum Psychopharmacol.

[127] Reilly JG, Ayis SA, Ferrier IN, Jones SJ, Thomas SH (2000). QTc-interval abnormalities and psychotropic drug therapy in psychiatric patients. Lancet.

[128] Yap YG, Camm AJ (2003). Drug induced QT prolongation and torsades de pointes. Heart.

[129] Tamene A, Sattiraju S, Wang K, Benditt DG (2010). Brugada-like electrocardiography pattern induced by severe hyponatraemia. Europace.

[130] Darpo B (2001). Spectrum of drugs prolonging QT interval and the incidence of torsades de pointes. Eur Heart J Suppl.

[131] Rouleau F, Asfar P (2001). Transient ST segment elevation in right precordial leads induced by psychotropic drugs: relationship to the Brugada syndrome. J Cardiovasc Electrophysiol.

[132] Civalier KA, Krahn LE, Agrwal N (2009). Repeated episodes of neutropenia triggered by mirtazapine. Psychosomatics.

[133] Andersohn F, Konzen C, Bronder E, Klimpel A, Garbe E (2009). Citalopram-induced bleeding due to severe thrombocytopenia. Psychosomatics.

[134] Lucena MI, Carvajal A, Andrade RJ, Velasco A (2003). Antidepressant-induced hepatotoxicity. Expert Opin Drug Saf.

[135] Azaz-Livshits T, Hershko A, Ben-Chetrit E (2002). Paroxetine associated hepatotoxicity: a report of 3 cases and a review of the literature. Pharmacopsychiatry.

[136] Pompili M, Tittoto P, Masciana R, Gasbarrini G, Rapaccini GL (2008). Acute hepatitis associated with use of paroxetine. Intern Emerg Med.

[137] Colakoglu O, Tankurt E (2005). Toxic hepatitis associated with paroxetine. Int J Clin Pract.

[138] Tabak F, Gunduz F, Tahan V, Tabak O, Ozaras R (2009). Sertraline hepatotoxicity: report of a case and review of the literature. Dig Dis Sci.

[139] Persky S, Reinus JF (2003). Sertraline hepatotoxicity: a case report and review of the literature on selective serotonin reuptake inhibitor hepatotoxicity. Dig Dis Sci.

[140] Friedenberg FK, Rothstein KD (1996). Hepatitis secondary to fluoxetine treatment. Am J Psychiatry.

[141] Cai Q, Benson MA, Talbot TJ, Devadas G, Swanson HJ, Olson JL, Kirchner JP (1999). Acute hepatitis due to fluoxetine therapy. Mayo Clin Proc.

[142] Otani K, Kaneko S, Tasaki H, Fukushima Y (1989). Hepatic injury caused by mianserin. BMJ.

[143] Hui CK, Yuen MF, Wong WM, Lam SK, Lai CL (2002). Mirtazapine-induced hepatotoxicity. J Clin Gastroenterol.

[144] Adetunji B, Basil B, Mathews M, Osinowo T (2007). Mirtazapine-associated dose-dependent and asymptomatic elevation of hepatic enzymes. Ann Pharmacother.

[145] Humayun F, Shehab TM, Tworek JA, Fontana RJ (2007). A fatal case of bupropion (Zyban) hepatotoxicity with autoimmune features: Case report. J Med Case Reports.

[146] Detry O, Delwaide J (2009). Fulminant hepatic failure induced by venlafaxine and trazodone therapy: a case report. Transplant Proc.

[147] Hull M, Jones R, Bendall M (1994). Fatal hepatic necrosis associated with trazodone and neuroleptic drugs. BMJ.

[148] Mclntyre RS, Panjwani ZD (2008). The hepatic safety profile of duloxetine: a review. Expert Opin Drug Metab Toxicol.

[149] Owen RT (2009). Agomelatine: a novel pharmacological approach to treating depression. *Drugs Today* (Bare).

[150] De Santy KP, Amabile CM (2007). Antidepressant-induced liver injury. Ann Pharmacother.

[151] Kostrubsky SE, Strom SC (2006). Inhibition of hepatobiliary transport as a predictive method for clinical hepatotoxicity of nefazodone. Toxicol Sci.

[152] McAllister-Williams RH, Baldwin DS, Haddad PM, Bazire S The use of antidepressants in clinical practice: focus on agomelatine. Hum Psychopharmacol.

[153] Lee WM (2003). Drug-induced hepatotoxicity. N Engl J Med.

[154] Twardowschy CA, Bertolucci CB, Gracia Cde M, Brandao MA (2006). Severe hyponatremia and the syndrome of inappropriate secretion of antidiuretic hormone (SIADH) associated with fluoxetine: case report. Arq Neuropsiquiatr.

[155] Wilkinson TJ, Begg EJ, Winter AC, Sainsbury R (1999). Incidence and risk factors for hyponatraemia following treatment with fluoxetine or paroxetine in elderly people. Br J Clin Pharmacol.

[156] Kirchner V, Silver LE, Kelly CA (1998). Selective serotonin reuptake inhibitors and hyponatraemia: review and proposed mechanisms in the elderly. J Psychopharmacol.

[157] Liu BA, Mittmann N, Knowles SR, Shear NH (1996). Hyponatremia and the syndrome of inappropriate secretion of antidiuretic hormone associated with the use of selective serotonin reuptake inhibitors: a review of spontaneous reports. Can Med Ass J.

[158] Barclay TS, Lee AJ (2002). Citalopram-associated SIADH. Ann Pharmacother.

[159] Nirmalani A, Stock SL, Catalano G (2006). Syndrome of inappropriate antidiuretic hormone associated with escitalopram therapy. CNS Spectr.

[160] Stovall R, Brahm NC, Crosby KM (2009). Recurrent episodes of serotonin-reuptake inhibitor-mediated hyponatremia in an elderly patient. Consult Pharm.

[161] Bavbek N, Kargili A, Akcay A, Kaya A (2006). Recurrent hyponatremia associated with citalopram and mirtazapine. Am J Kidney Dis.

[162] Koelkebeck K, Domschke K, Zwanzger P, Hetzel G, Lang D, Arolt V (2009). A case of non-SIADH-induced hyponatremia in depression after treatment with reboxetine. World J Biol Psychiatry.

[163] Rosner MH (2004). Severe hyponatremia associated with the combined use of thiazide diuretics and selective serotonin reuptake inhibitors. Am J Med Sci.

[164] Sommer BR, Fenn HH, Ketter TA (2007). Safety and efficacy of anticonvulsants in elderly patients with psychiatric disorders: oxcarbazepine, topiramate and gabapentin. Expert Opin Drug Saf.

[165] Battaglino R, Fu J, Spate U, Ersoy U, Joe M, Sedaghat L, Stashenko P (2004). Serotonin regulates osteoclast differentiation through its transporter. J Bone Miner Res.

[166] Williams LJ, Henry MJ, Berk M, Dodd S, Jacka FN, Kotowicz MA, Nicholson GC, Pasco JA (2008). Selective serotonin reuptake inhibitor use and bone mineral density in women with a history of depression. Int Clin Psychopharmacol.

[167] Bolton JM, Metge C, Lix L, Prior H, Sareen J, Leslie WD (2008). Fracture risk from psychotropic medications: a population-based analysis. J Clin Psychopharmacol.

[168] Ziere G, Dieleman JP, van der Cammen TJ, Hofman A, Pols HA, Strieker BH (2008). Selective serotonin reuptake inhibiting antidepressants are associated with an increased risk of nonvertebral fractures. J Clin Psychopharmacol.

[169] Dodd S, Hodge J, Williams L, Berk M, Nicholson G (2008). The effect of SSRIs on osteoclast formation and functioning. Poster presented at the 26th Collegium Internationale Neuro-Psychopharmacologicum Congress, Munich, 13-17 July 2008. Int J Neuropsychopharmacol.

[170] Hemeryck A, Belpaire FM (2002). Selective serotonin reuptake inhibitors and cytochrome P-450 mediated drug-drug interactions: an update. Curr Drug Metab.

[171] van Harten J (1995). Overview of the pharmacokinetics of fluvoxamine. Clin Pharmacokinet.

[172] Mandrioli R, Forti GC, Raggi MA (2006). Fluoxetine metabolism and pharmacological interactions: the role of cytochrome p450. Curr Drug Metab.

[173] Spina E, Santoro V, D'Arrigo C (2008). Clinically relevant pharmacokinetic drug interactions with second-generation antidepressants: an update. Clin Ther.

